# Knowledge Graph Analysis of Human Health Research Related to Climate Change

**DOI:** 10.3390/ijerph17207395

**Published:** 2020-10-11

**Authors:** Yating Zhao, Jingjing Guo, Chao Bao, Changyong Liang, Hemant K Jain

**Affiliations:** 1 The School of Economics and Management, Hefei Normal University, Hefei 230601, China; 2 The School of Management, Hefei University of Technology, Hefei 230009, China; veralien@163.com (J.G.); yancey1246@163.com (C.B.); cyliang@163.com (C.L.); 3 College of Business, the University of Tennessee at Chattanooga, Chattanooga, TN 7403, USA; hemant-jain@utc.edu

**Keywords:** climate change, human health, public health, knowledge evolution, visual analysis, hotspots

## Abstract

In order to explore the development status, knowledge base, research hotspots, and future research directions related to the impacts of climate change on human health, a systematic bibliometric analysis of 6719 published articles from 2003 to 2018 in the Web of Science was performed. Using data analytics tools such as HistCite and CiteSpace, the time distribution, spatial distribution, citations, and research hotspots were analyzed and visualized. The analysis revealed the development status of the research on the impacts of climate change on human health and analyzed the research hotspots and future development trends in this field, providing important knowledge support for researchers in this field.

## 1. Introduction

The global climate is changing, which is resulting in global warming, melting glaciers, rising sea levels, and extreme weather patterns [1]. One of effects of climate change is the increase of infectious diseases (e.g., allergens, inflammatory bowel disease (IBD), and infectious gastroenteritis (IG)) [2,3]. The World Health Organization predicts that the annual number of deaths due to climate change will reach 250,000 between 2030 and 2050 [4,5]. The IPCC (Intergovernmental Panel on Climate Change) Fourth Assessment Report of the United Nations Intergovernmental Panel on Climate Change also pointed out that the health status of millions of people around the world will be affected by climate change, including an increase in the number of undernourished people [6]. Diarrheal diseases caused by extreme weather events will lead to increased deaths, illnesses, and injuries. As the concentration of ground-level ozone associated with climate change increases, the incidence of cardiopulmonary disease will increases and the spatial distribution of certain infectious diseases changes [7].

The impact of climate change on population health is not only related to climate change factors, but is also affected by many other factors that are impacted by climate, such as the degree of socioeconomic development, the regional geographic environment, the availability of resources, infrastructure construction, and the importance of local government [8]. For example, for areas with frequent hurricanes, if an early warning mechanism is established, the population has a strong sense of disaster prevention and the damage is limited if infrastructure is relatively complete. If there are sufficient shelters in the area, the extreme weather will have less impact on the health of local residents. Assessing the impact of climate change on population health is becoming increasingly important. Climate change has become a major environmental and public health problem facing countries all over the world. Working out how to assess the health risks caused by it, how to mitigate the risks, and how to improve public adaptation have become important research topics for environmental and public health workers [9].

The research on human health effects driven by climate change has attracted the attention of a large number of researchers globally [10]. Most of the research has focused on the following aspects. First, the research has focused on the health effects of global climate change. Scholars such as Arbuthnott, Barbara, Snow, Lichtveld, Myers, and Sheng have focused on how climate change affects human health and where it affects it. Second, the research has focused on how to assess the impacts of climate change on population health. Refs. [11,12,13,14,15,16] established a comprehensive assessment method for a vulnerability index (VI) and combined it with geographic information systems to visually display the regional distribution of climate change and health vulnerability. Various models are used to assess the health effects of climate change on related diseases [17]. Third, the research has focused on prevention and control measures. To reduce the health risks of heat waves, many researchers have studied heat health warning systems, aiming to convey the most accurate weather information to the public at the fastest speed and to take corresponding preventive measures to minimize public losses [18]. Stewart and Hytiris suggest that improving infrastructure, such as drainage, will reduce the human health impacts of extreme weather.

In summary, although there have been many research achievements in the field of human health research driven by climate change, there are still many gaps. First, no scientific research paper has quantitatively examined the development status of the field from the perspective of bibliometrics. Second, no analysis has been carried out from a full-time publication frame perspective. Third, there is a lack of studies that analyze global literature. Due to the lack of a global perspective that can provide researchers in related fields with a comprehensive picture of the current situation of climate change and health research, the overall understanding of this field is limited. This makes it difficult to properly allocate resources in this field, which to a large extent leads to inefficient research.

In order to fill these research gaps, this paper analyzes 6719 documents related to climate change and health research obtained from the Web of Science database from the perspectives of bibliometrics and information visualization. This paper reveals the knowledge structure and evolution process in this field of research, discovers popular research issues, explores future trends and development directions, and provides panoramic knowledge support for researchers in this field.

The rest of the paper is organized as follows. The next section introduces the methodology used in this study. Section 3 and Section 4 introduce the knowledge map of the temporal and spatial analysis. Section 5 presents the knowledge base analysis. Section 6 presents the research hotspot analysis. The last section concludes the study and presents future directions of research.

## 2. Methodology

### 2.1. Data Source

The data used in the study were collected from Web of Science, which is an authoritative platform for scientific documents worldwide [19]. Web of Science (WOS) has a collection of scientific literature from more than 9000 academic journals, which ensures the representativeness and authority of the literature sources. The data in the platform are updated once a week, which ensures the timeliness of the literature. This paper uses the core data collection from the Web of Science (WOS) database ordered by Hefei University of Technology as the source of the literature. There are four databases: SCI-E, CPCI-S, CCR-E, and IC. The search strategy we used is #1 AND #2, where ”#1” refers to a series of keywords related to climate change (e.g., “climate change” and “climatic variation” and “climatic change”), while ”#2” indicates the health keyword “health”. Yao compared academic literature sources, such as monographs and research reports, and found that papers published in journals are relatively more continuous, sensitive, and directly related to the academic field [20]. Therefore, this paper only selects scientific literature of the “article” type. The retrieval period is from 2003 to 2018 and the retrieval time was 21 June 2018. A total of 6719 documents were collected.

### 2.2. Toolkits

Bibliometrics is a cross-discipline field containing methods that quantitatively analyze all knowledge carriers in the academic literature as the research object by using mathematics, statistics, and philology. Using bibliometrics, we can mine the tacit knowledge in the literature and reveal the hotspots and future trends of a discipline [21]. The study of literature quantification originated in the early 20th century, when books and journal articles were statistically classified by using quantitative methods. It was not until the 1960s that the relevant researchers introduced metrology into statistics and began the study of bibliometrics. At present, bibliometric analysis has become an important research paradigm in information management, information science, and library science. The advantage of the bibliometric analysis method is that it can mine the hidden knowledge in the literature from a large amount of literature data by using data analysis tools, and it can also use statistical methods to analyze and summarize the development direction and predict the future trends of a certain discipline [22,23,24].

The concept of scientific knowledge maps can be traced back to a seminar organized by the national academy of sciences in 2003. With the development of information visualization, various scientific knowledge mapping tools have emerged. Citespace is one of the popular information visualization tools, which was developed by Professor Chen of Drexel University [25]. It is based on co-citation analysis theory and a path finding network algorithm (PFNET). It calculates and analyzes literature in a specific field, explores key paths and knowledge turning points in the evolution of the discipline domain, and explores potential dynamic mechanisms of discipline evolution and frontiers of discipline development through a series of visualization map analyses. We used CiteSpace and Histcite for data analysis and visualization based on 6719 literature documents. The results revealed national institution and collaboration networks, knowledge evolution, research hotspots, and future trends in the interdisciplinary research field of climate change and human health.

## 3. Knowledge Map of Temporal and Spatial Analysis

**Figure 1:**
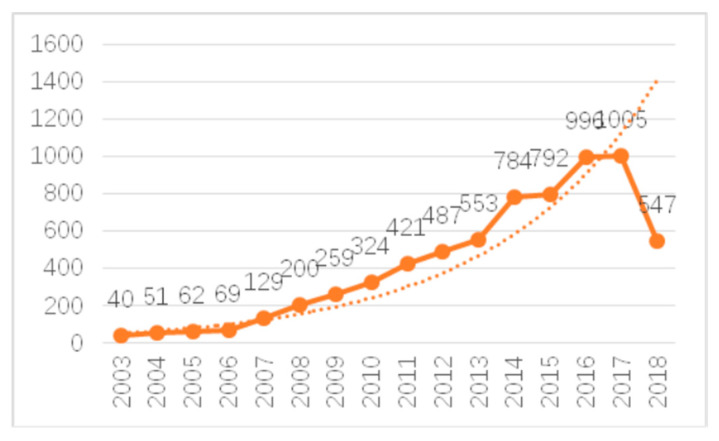
Annual number of published articles.

The change in the number of published articles in a specific field can directly reflect the development of the field and help researchers to intuitively grasp the development status and trends in that field. In order to investigate the research in the field of climate change and health, we compiled statistics on the number of articles published during 2003–2018 and obtained the annual trend shown in Figure 1.

Research on climate change and human health originated in the early 2000s. Climate change effects such as global warming have become an indisputable fact. Social development and progress and quality of life improvements have become increasingly important. People are increasingly managing their own health, for example by living in areas with cleaner air. From 2003 to 2006, the research on climate change and health was in the initial stage of development, with immature theories and methods, few scientific research results, and no more than 100 academic articles published each year. From 2007 to 2017, the number of scientific articles published rose in almost a straight line and was much higher than the exponential forecast curve. The number of scientific articles is expected to continue to grow since we retrieval time is June 2018. It is clear that climate change and health research as a whole is booming.

**Figure 2:**
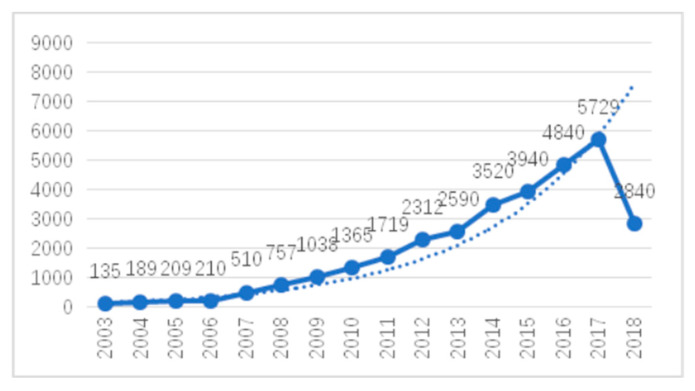
Annual number of authors.

In order to investigate the input of scientific researchers in the field of climate change and health, the number of scientific researchers involved over the years was calculated and the trend of annual author input was obtained, as shown in Figure 2. It can be seen that the trend of annual author input is consistent with the trend of scientific literature quantity, which generally shows exponential growth before 2006 and linear growth after 2007. There were 135 authors in the field in 2003 and 5729 in 2017. In fourteen years, the number of authors has increased by about forty-two times. The data for 2018 are not yet complete and were not used in the comparison. It can be seen that with the attention given from all sectors of society, more authors are devoting to studies on climate change and health.

**Figure 3:**
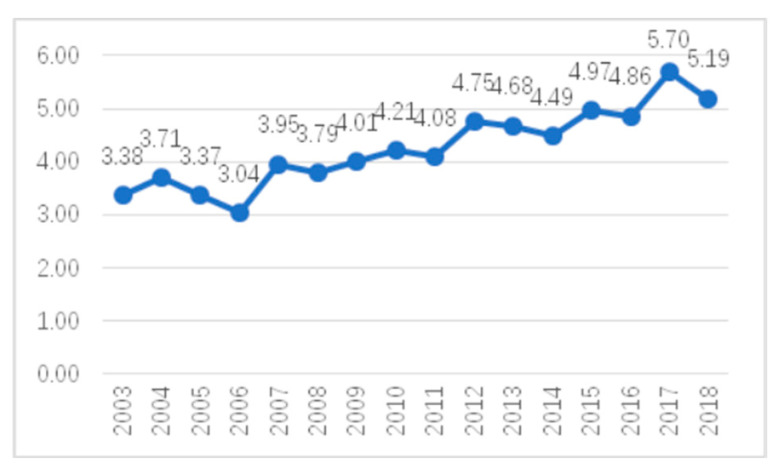
Average number of co-authors per article.

In order to investigate the input–output ratio of researchers in the field of climate change and health research, the number of participants in a single paper was calculated and the trend for the average number of participants in a single paper was obtained, as shown in Figure 3. The average number of participants in a single paper increased from 3.38 in 2003 to 5.7 in 2017. Later, a trend of increasing cooperation among countries and institutes was also found. It can be seen that the degree of collaboration of the authors in the field of climate change and health has increased, which guarantees the quality of the papers to some extent and also reflects the attention paid to the research in this field.

## 4. Space Distribution Map

### 4.1. Institutional Distribution

The core academic groups and institutions in the field of climate change and health are shown in Table 1. The table shows the information related to the top 10 organizations involved in the research. Australian National University was slightly ahead of the Chinese Academy of Sciences. Subsequently, three institutions, Harvard University, University of Queensland, and James Cook University, published 126, 110, and 109 articles, respectively. The subsequent five institutions were closely matched in their numbers of publications, indicating that the research on climate change and health was of great concern to international academic institutions. Most of the institutions that published scientific literature in the top 10 were in developed countries, with the United States accounting for 40%, followed by Australia with 30%, then the UK, Canada, and China with 10% each. This shows that developed countries have done more studies than developing countries in the area of climate change and health.

It can be seen in Table 1 that although the Chinese Academy of Sciences published the second largest number of articles, their local citation score (LCS) ranked last among the top 10 institutions, indicating that the institution had a weak influence. In contrast, although Columbia University, London School of Hygiene and Tropical Medicine, and US EPA (United States Environmental Protection Agency) rank low in their numbers of published articles, their LCS scores are 725, 760, and 317, respectively. This suggests that these institutions have the clout needed to make ground breaking innovations in the scientific research on climate change and health.

**Figure 4:**
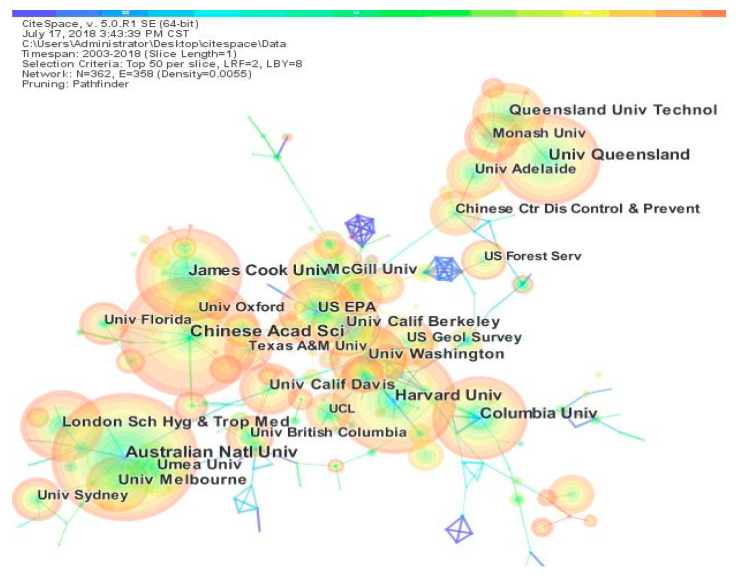
Institutional collaboration network. Note: Queensland Univ Technol (Queensland University of Technology); Monash Univ (Monash University); Univ Queensland (The University of Queensland); Univ Adelaide (The University of Adelaide); Chinese Ctr Dis Control&Prevent (China Center for Disease Control and Prevention), US Forest Serv (US Forest Service); James Cook Univ (James Cook University); McGill Univ (McGill University); Univ Oxford (University of Oxford); US EPA (United States Environmental Protection Agency); Univ Florida (University of Florida); Univ Calif Berkeley (University of California, Berkeley); Chinese Acad Sci (Chinese Academy of Sciences); US Geol Survey(The United States Geological Survey); Texas A&M Univ (Texas A&M University); Univ Washington (University of Washington), Univ Calif Davis (University of California, Davis); Harvard Univ (Harvard University); UCL (University College London); Columbia Univ (Columbia University); London Sch Hyg&Trop Med (London School of Hygiene & Tropical Medicine); Univ British Columbia (The University of British Columbia); Australian Natl Univ (Australian National University); Ume a Univ (university of umeå); Univ Melbourne (The University of Melbourne); Univ Sydney (The University of Sydney).

Since research cooperation is an important way to enhance the overall research strength and realize the complementary advantages of scientific research resources and knowledge sharing [27], the degree of research cooperation is one of the indicators reflecting the research status of institutions in the field. To study institutional cooperation in the field of climate change and health, a network of institutional cooperation in the field was established using Citespace, as shown in Figure 4. The size of a node is proportional to the number of articles published by an institution, the thickness of a line between nodes is proportional to the number of collaborative articles between institutions, and different colors indicate the year of cooperation between institutions. As can be seen from the chart, the cooperation between major research institutions is relatively close. As can be seen from the color of the annular shapes, in recent years, increasing numbers of posts have been issued by major institutions at home and abroad. The cool shades (green) in the picture represent the early stages of climate change and health development, which are gradually being overwhelmed by the recent agencies in the cooperative network. This phenomenon indicates that the development of this field is currently occurring rapidly, and increasing numbers of scientific research articles are being published (indicated by the thickness of the outer warm layers of the year rings).

### 4.2. National and Regional Distribution

As shown in Table 2, in terms of the number of publications, the USA has an advantage—the number of scientific articles for the USA is 2420, putting it in first place. The next two countries, Australia and the UK, published 954 and 928 articles, respectively. In terms of the frequency of citations, the USA, Australia, and the UK topped the list with 5236, 2365, and 2518 citations, respectively. This shows that the United States, Australia, and the United Kingdom attach importance to the impacts of climate change on health and have invested a lot in academic research, actively carrying out research and exploring solutions and contributing greatly in this field. China ranks fourth with 642 articles, but the frequency of citations is far lower than that of Canada. This shows that China has relatively low influence in this field and that the quality of scientific research achievements still needs to be further improved. In addition to the top five countries, other major countries and regions also deserve attention, such as Italy, Spain, and Sweden. Although these countries have relatively fewer publications, their LCS scores have exceeded 500, which indicates that the quality of academic papers published in these countries is relatively high. Of the top 10 countries, all but China are developed countries. Developed countries usually have relatively complete and long time series of monitoring data, so a large number of high-quality articles have been published. Most of the monitoring data in developing countries are not perfect, so there is less research and scientific literature published in these countries. Therefore, developing countries should establish and perfect meteorological health monitoring systems that are consistent at temporal and spatial scales. The collection of data from longer time series is the basis of climate change and health research.

**Figure 5:**
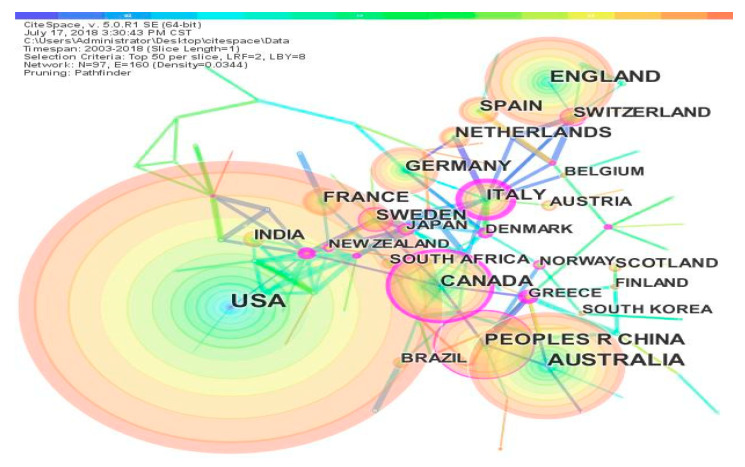
National collaboration network.

As shown in Figure 5, the country and region collaboration network map was created using CiteSpace. The top color bar represents the year (ranging from 2003 (left) to 2018 (right)), the size of the color rings represents the number of publications, the color represents the year of publication, the lines between the year rings represent the cooperative relationship between countries and regions, and different colors represent different cooperative years. The map clearly shows the national and regional cooperation in research on climate change and health. First of all, it can be seen that the warm colors (red, yellow, orange, etc.) are the most prevalent, while the cold colors (blue, indigo, green) are less prevalent. This indicates that research in the field of climate change and health has mainly occurred in recent years and that less work was published in the early stages. With the development of this field, more countries and regions have become involved. In terms of centrality, Italy’s maximum value is 0.54, followed by Canada’s at 0.22, then 0.17 for Sweden and 0.16 for China, which are marked in purple in Figure 5. Centrality describes the importance of the node to other nodes. This shows that these countries occupy an important position in the field of climate change and health. In total, there are 97 countries and regions and 160 cooperation links between countries. In general, there is close cooperation between countries and regions in the field of climate change and health research. Climate change is a major challenge facing the world today. Countries need to work together and strengthen these cooperative relationships. Countries with different development stages and scientific and technological levels should make joint efforts toward climate change based on the principle of common but differentiated responsibilities [28].

### 4.3. Author Distribution

The core writers group refers to the collection of authors with a large number of published articles and great influence in the subject area. According to the distribution law of the authors in the subject area, Price’s law is M = 0.749(NMax)/2, where NMax refers to the number of authors who have the most published articles in the field, while scholars who publish more than M are the core authors in this field [29]. Based on the analysis of the document using Histcite, the author with the largest number of documents is Tong with 51 articles; therefore, NMax = 51. Thus, based on Price’s law, M = 0.749 × 51/2 = 19.1. This shows that the authors with more than 20 articles are the core authors in the field of climate change and health. There are 20 authors in the core group. Table 3 shows the core authors and their citation information.

**Figure 6:**
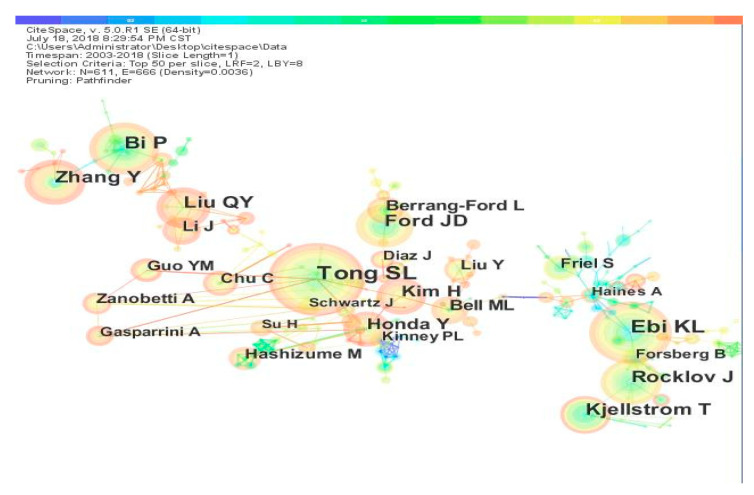
Co-author network.

The network of authors in the field of climate change and health is shown in Figure 6. The relationships between academic researchers are characterized by the number of nodes, the distribution, and the number of links. The number of nodes indicates that the development of this field involves a large number of researchers. The distribution is heavily concentrated and there are many links, which shows the cooperation between authors. In addition, the authors’ cooperation links are mostly warm colors, indicating that the core authors in this field have become more closely connected in recent years.

### 4.4. Journal Distribution

By analyzing the distribution of journals, researchers can understand which are the core journals and marginal journals, the spatial distribution of published articles, and the preferred journals in this field in order provide a reliable reference source for future researchers to conduct in-depth research in this field. Core journals are a dense source of information. They have reference value related to the determination of selected work, collection work, reader work, information service work, and so on [30]. Table 4 shows the 10 journals with the most published articles. A total of 1193 articles were published in these journals, accounting for 17.76% of the total publications in this field. Among them, PLOS One, International Journal of Environmental Research and Public Health, Science of the Total Environment, Climatic Change, and Environmental Health Perspectives are the top five journals. PLOS One published the most articles, but the citation frequency LCS = 0. This indicates that PLOS One contributes a lot to climate change and health research, but its actual influence is relatively low and the quality of scientific research achievements still needs to be further improved. On the contrary, Environmental Health Perspectives, although being relatively small in its number of articles, has the highest LCS of 1212. This indicates that the academic papers published by Environmental Health Perspectives are of high quality and have great influence.

## 5. Knowledge Base Analysis

In order to understand the knowledge base in the field of climate change and health, Citespace III was used for literature co-citation analysis and a literature co-citation network was obtained. A co-citing network is a knowledge network formed under specific circumstances when two articles are cited simultaneously by a third article or multiple different articles. Co-citation analysis expresses the relationship between documents by the frequency they are cited by other documents at the same time; that is, two documents are simultaneously cited by another article. The higher the frequency of citations, the closer the relationship between the two, i.e., the more similar the academic background of the two documents. Fundamentally, when certain documents, journals, or academic groups are repeatedly quoted by peers, the knowledge carriers that are cited are recognized by the scientific community, which are formed to create a scientific paradigm. This paradigm relationship can be visualized by analysis of the co-citation network in the literature. The scientific paradigm refers to the formation and establishment of a set of conceptual systems and analytical methods that are generally accepted and used by people in a certain subject area as a communication idea [31]. Therefore, through the literature co-citation network, the knowledge base in the field of climate change and health can be specifically demonstrated.

**Figure 7:**
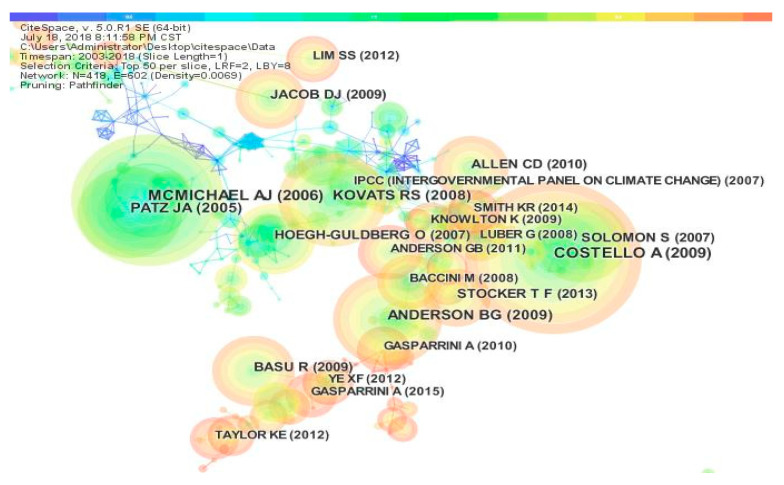
Article co-citation network.

Figure 7 shows the literature co-citation network. Each node represents a referenced article. The lines between pairs of nodes represent common reference relationships. The thickness of the lines represents the frequency of the common references. The number of network nodes is 418, the number of connections between nodes is 602, and the density of the network is 0.0069. In the literature citation network, Costello published an article in The Lancet journal entitled “Managing the Health Effects of Climate Change: Lancet and University College London Institute for Global Health Commission”, which was the most cited article at 220 citations [32]. Costello is connected with Myers, Mccmhaha, and Ford, and the connection line with Ford is the thickest [14], indicating that the co-citation relationship between them is strong. This also shows that the scientific literature published by Costello is more relevant to the scientific literature published by Ford, and that the topics in the scientific literature are similar. Gasparrini published an article in The Lancet titled “Mortality Risk Attributable to High and Low Ambient Temperature”. A multi-county observational study was also cited frequently, reaching 64 citations. Gasparrini is connected with Hajat, Guo, and Phillips, and the connection lines with Hajat and Phillips are strong, which indicates that Gasparrini has a strong correlation with these two scientific articles and has a similar theme. On the whole, the literature co-citation network in the field of climate change and health research is scattered and less connected, which is related to the integration of the two fields of climate change and health and the insufficient development time, meaning there are no complete co-citation relationships.

**Figure 8:**
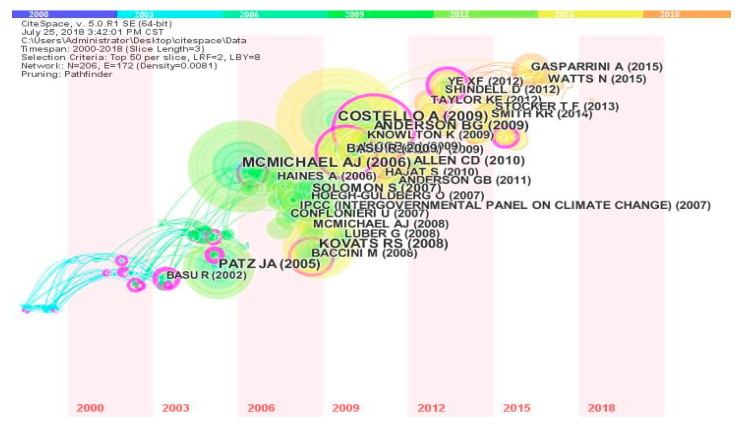
Article co-citation time zone diagram.

The literature co-citation time zone diagram is shown in Figure 8. It shows that important scientific literature in each period of climate change and health research can be seen, and these scientific articles constitute an innovative path in this field. Highly cited articles on climate change and health are widely distributed between 2009 and 2011, and the most influential references are also distributed over this time interval. This indicates that breakthroughs were made in academic research in the field of climate change and health between 2009 and 2011. For example, Costello pointed out that climate change is the biggest global health threat in the 21st century. The impact of climate change on health will aggravate inequality between rich and poor. The new public health campaigns will increase publicity to reduce climate change and other important views [32], which will play a significant role in promoting the importance of climate change and health. After 2009, not only did people pay more attention to climate change and health issues, but correspondingly the academic and political research on climate change and health increased. The United States, a large country outside the international agreement for greenhouse gas emission reductions under the Kyoto Protocol, is facing strong international pressure. In 2009, the US House of Representatives passed the US Clean Energy and Security Act, marking an important step in climate change legislation [33]. As a major economy, the second largest emitter of greenhouse gases, and the world’s largest per capita emitter of greenhouse gases, the active participation of the United States is critical in addressing global climate change. Table 5 shows the top ten published papers in terms of citation frequency. These scientific articles constitute the knowledge base of research in the field of climate change and health. In the future, researchers will carry out research on new hot issues along these knowledge base paths and continue the extension of innovation.

In summary, these important researchers and important scientific literature in this field have promoted the development of climate change and health research and played an important foundational role in the development of relevant theories in this field.

## 6. Research Hotspot Analysis

Keywords summarize the major content, academic thoughts, and principal research methods of the researchers, which are the core and essence of the literature. Frequently appearing keywords are often used to identify major topics in a research field. By analyzing the keywords, we can intuitively grasp the main research content of a paper and even the overall research situation of a research field [42]. In order to understand the current knowledge structure and hot topics in the field of climate change and health, we extracted and calculated the frequency of keywords in 6719 articles. Table 6 shows the top 20 keywords and the co-occurrence frequency.

The high frequently occurring topics in this field are climate change and health. This result is consistent with the research object of this paper. This shows that the core of the research in this field must be related to climate change and health. It can be seen from the table that high-frequency keywords such as temperature, air pollution, and heat wave have become the hot issues of climate change and health research. Among them, global warming is a prominent problem in climate change. There is a relatively high co-occurrence frequency of keywords such as adaptation, vulnerability, model, risk, management, variability, and exposure. These keywords represent the techniques or methods in the field of climate change and health. Many researchers believe that mathematical models can be applied to climate change research to assess the health risks [43]. Due to differences in the levels of economic development and population adaptability in various regions, the impacts of climate change on population health is uneven. Generally speaking, climate change is most harmful to vulnerable populations in vulnerable areas [44]. Therefore, it is important to carry out a climate change health vulnerability assessment. Vulnerability is a comprehensive measure of the extent to which a system is threatened by the adverse effects of climate change and is a function of climate risk exposure levels, system sensitivity, and adaptive capacity. At present, academics often use vulnerability to reflect the extent to which climate change adversely affects an area or population. Mitigation and adaptation are two major strategies for addressing climate change [45]. At present, climate change mitigation has been given special attention internationally and a large amount of resources have been invested in this topic. Even if there is international agreement to take action to reduce greenhouse gas emissions, warming will continue to occur because of the long half-life of greenhouse gases. Therefore, adaptation to climate change is an important complement to mitigation of climate change and is a hot topic in the field of climate change and health. From the perspective of public health, adaptation to climate change refers to measures taken to prevent health risks caused by climate change. Whether a region or a population can adapt to climate change well depends on its ability to adapt [46]. A region’s ability to adapt is also affected by its socioeconomic resources, technological level, information and skills, infrastructure, social system, degree of social equity, and existing disease patterns. The IPCC believes that strengthening public health infrastructure is the most important and effective adaptation strategy [47], which includes carrying out public health training projects, establishing more effective monitoring and emergency response systems, and carrying out sustainable prevention and control projects. Therefore, public health is also a hot topic for scholars in the field of climate change and health. China has a fragile ecological environment, long coastline, and low per capita resource occupancy. It is extremely vulnerable to the adverse effects of climate change, so it is facing more severe challenges in tackling it. With the rapid development of China’s economy, environmental governance has been strengthened, its ability to adapt to the environment has been enhanced, and great achievements have been made. Therefore, climate change in China has become a hot topic for scholars in the field of climate change and health.

**Figure 9:**
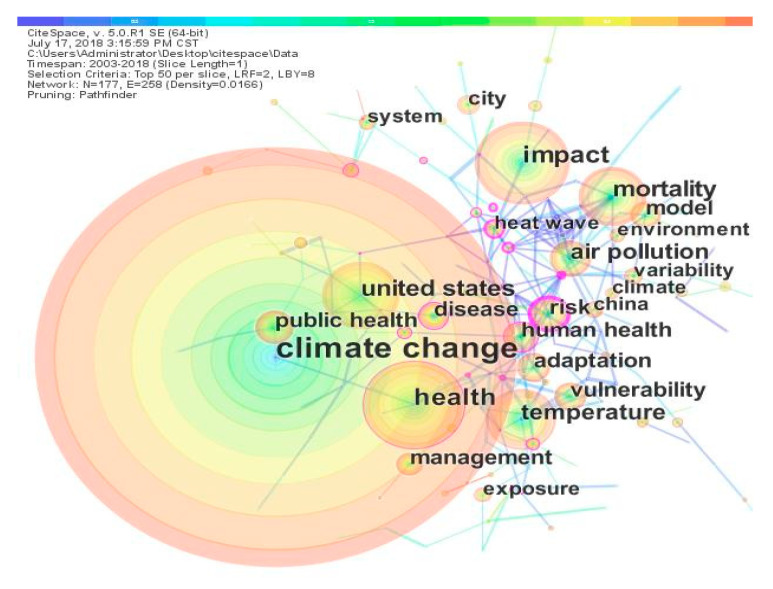
Keywords co-occurrence network.

The idea of co-word analysis is that if two keywords appear together in one article, then the two topics they represent are related. The higher the degree of co-occurrence, the stronger the relationship. The two subject words are constructed into a common word network, and the distance between the nodes indicates the relationship between the subject words. By cluster analysis of subject words, several subject words can be gathered together to constitute a research subject field [48]. Our study builds a keyword co-occurrence matrix using CiteSpace and draws a keyword co-occurrence network, as shown in Figure 9. Each node in the graph represents a keyword. The size of a node is proportional to the co-occurrence frequency. The connections between the nodes represent the co-occurrence relationships between pairs of keywords in the same document. Different colors indicate the year the keywords appear together. The number of network nodes is 177, the number of connections between nodes is 258, and the density of the network is 0.0166. According to this figure, there are strong connections between the keywords and the entire network is densely connected. This shows that most of the papers published in this research field are multi-topic research papers.

## 7. Conclusions and Future Trends

This paper summarizes the development status and knowledge base of the research in the field of climate change and health and analyzes the hot research issues, providing an important reference to allow follow-up researchers to grasp the development of science and research issues in this field.

First, from 2003 to 2006, the annual number of published articles and the input curve for the annual number of authors showed exponential growth trends. From 2007 to 2017, the annual number of published articles and the input curve for the annual number of authors grew rapidly and were much higher than the exponential forecast curve. On the whole, the number of articles published and the annual input of authors showed upward trends from 2003 to 2017, while the growth trends in recent years were even more rapid. The average number of participants in a single paper reached 4.75, indicating more cooperation among the authors in this field.

Second, in terms of spatial distribution, cooperation between agencies is more frequent. The major contributing institutions are Australian National University, Chinese Academy Sciences, Harvard University, University of Queensland, James Cook University, and Columbia University. Cooperation between countries and regions is also closer. The major contributing countries are the USA, Australia, the UK, Peoples Republic of China, and Canada. It should be noted that although there are many publications in China, the volume and centrality of the paper are low and the quality of the paper should be emphasized. The cooperation between the authors is relatively close. The main contributors are Tong, Ebi, Bi, Zhang, and Ford. PLOS One, International Journal of Environmental Research and Public Health, Science of The Total Environment, Climatic Change, and Environmental Health Perspectives are the top five journals in the research field.

Third, the literature co-citation network is not dense enough. Costello published an article in The Lancet journal entitled “Managing the Health Effects of Climate Change: Lancet and University College London Institute for Global Health Commission”, which was the most cited article with 220 citations. In general, the distribution of the co-citation network is relatively dispersed, with few connections. A relatively complete and mature co-citation network system has not been formed.

Fourth, in the keyword co-occurrence network, there are more links between the keywords and the whole network is more densely connected. It is shown that most of the papers published in the field of climate change and health research are multi-thematic. The research hotspots are diverse. The major climate change hotspots are temperature, air pollution, and heat waves. The technical method hotspots are adaptability, vulnerability, model, risk, and management.

Based on the results of the bibliometric analysis and a systematic review, we summarize the following development trends in the field of climate change and health.

The first trend is of human health vulnerability assessment under the influence of climate change. From the perspective of research hotspots in this field, vulnerability assessment has always been an important topic for guiding human beings to adapt to future climate change, and has important theoretical and practical value. Through vulnerability assessment, regions and populations that are sensitive to climate change can be identified, and adaptation measures can be taken to protect the health of vulnerable populations and reduce the impact of climate change on health [49]. At present, many researchers have carried out research on vulnerability to climate change, but there are few relevant research results in the field of health. In the field of health, relevant research should be carried out as soon as possible to develop methods and tools suitable for vulnerability assessment in the field, to establish an indicator system, and to collect basic data. The research techniques should be expanded to assess the impacts of climate change on the health of regional populations more scientifically and to identify vulnerable populations, discover obstacles to the population adapting to climate change, and provide a scientific basis for government departments to formulate relevant policies.

The second trend is of health risk assessment and early warnings based on climate change. Due to the complexity of the health effects caused by climate change, we have not fully realized the seriousness and urgency of the health risks of climate change and the need to take responsive actions [50]. Climate change can affect human health in direct or indirect ways, such as heat waves and extreme weather. These risks are interwoven with evolving socioeconomic conditions, medical technology, demographics, environmental conditions, and other factors that determine health. Health risk models are used to reflect how health determinants and climate change move in time and space, to reduce or avoid the adverse effects on health, and to guide climate policy and action [51]. Many organizations are modeling the effects of climate change locally, regionally, or globally. Most models focus on specific sectors, such as agriculture, energy, or the economy, and there are very few models for human health. Health risk models based on climate change should be the focus of future research [52,53].

The third trend is of strengthening communication and cooperation. On the one hand, exchanges and cooperation with other disciplines should be strengthened, especially in the field of meteorology, by understanding the characteristics of future regional climate change, obtaining simulation data on the future climate based on climate change scenarios, and conducting relevant research on climate events that may be encountered in the future. On the other hand, we must strengthen international cooperation. Since the signing of the United Nations Framework Convention on Climate Change in 1992, countries around the world have been working together to tackle climate change for more than 20 years [54,55]. Climate change has changed from a purely scientific problem to a complex political one involving the sustainable development of all mankind. Since the beginning of the 21st century, the developed countries have been mired in an economic crisis, while the emerging economies have gradually increased their economic strength and international voice. In addition, the global pattern of greenhouse gas emissions has greatly changed since the 1990s. Therefore, developed countries, emerging economies, and developing countries should strengthen their dialogue and exchanges on the principle of “common but differentiated responsibilities and corresponding capabilities”. Concepts such as carbon space allocation, capital and technology transfer, and academic and scientific research need to be promoted to jointly tackle the challenges posed to human health by global climate change.

## Figures and Tables

**Table 1:** List of the top 10 institutions with their numbers of published articles.

Institution	Recs	LCS	GCS
Australian National University	150	902	4487
Chinese Academy of Sciences	140	58	1806
Harvard University	126	711	5370
The University of Queensland	110	272	2632
James Cook University	109	180	3116
Columbia University	102	725	4606
London School of Hygiene & Tropical Medicine	92	760	4618
University of Washington	89	81	2224
McGill University	88	259	2033
United States Environmental Protection Agency	88	317	2153

Note: Recs (records) refers to the number of articles in the local database; LCS (local citation score) refers to the citation frequency of an article in the local database; GCS (global citation score) refers to the citation frequency of an article in the Web of Science database [26].

**Table 2:** List of the top 10 countries and regions, with numbers of published articles.

Country	Recs	LCS	Centrality
USA	2420	5236	0.04
Australia	954	2365	0.08
UK	928	2518	0
China	642	853	0.16
Canada	641	1491	0.22
Germany	456	486	0.09
Italy	342	748	0.54
France	299	410	0.07
Spain	279	599	0.04
Sweden	275	679	0.17

Note: Recs (records) refers to the number of articles in the local database; LCS (local citation score) refers to the citation frequency of an article in the local database.

**Table 3:** Authors with 20 or more published articles.

Author	Recs	LCS	GCS
Tong, S.L.	51	311	1390
Ebi, K.L.	49	312	1068
Bi, P.	39	267	751
Zhang, Y.	39	84	555
Ford, J.D.	36	184	700
Rocklov, J.	35	219	897
Kjellstrom, T.	33	289	837
Liu, Q.Y.	33	51	186
Kim, H.	28	261	735
Bell, M.L.	25	437	1522
Honda, Y.	25	225	648
Kinney, P.L.	25	365	944
Schwartz, J.	24	322	1904
Berrang-Ford, L.	22	91	330
Guo, Y.M.	22	192	694
Haines, A.	22	480	2704
Li, J.	22	5	132
Chu, C.	21	76	209
Friel, S.	21	119	498
Zanobetti, A.	21	221	1065
Hansen, A.	20	137	346
Hashizume, M.	20	203	655

Note: Recs (records) refers to the number of articles in the local database; LCS (local citation score) refers to the citation frequency of an article in the local database; GCS (global citation score) refers to the citation frequency of an article in the Web of Science database [26].

**Table 4:** Top 10 journals with the most published articles.

Journal	Recs	LCS	GCS
PLOS One	216	0	2997
International Journal of Environmental Research and Public Health	180	378	1331
Science of the Total Environment	169	321	2369
Climatic Change	144	437	3136
Environmental Health Perspectives	96	1212	4295
International Journal of Biometeorology	91	425	1794
Journal of Cleaner Production	81	26	827
Environmental Research Letters	73	0	853
Environmental Research	72	293	1248
Environmental Science and Technology	71	145	1810

Note: Recs (records) refers to the number of articles in the local database; LCS (local citation score) refers to the citation frequency of an article in the local database; GCS (global citation score) refers to the citation frequency of an article in the Web of Science database [26].

**Table 5:** Literature citation frequency ranking.

Year	Authors	Articles	Journal
2009	COSTELLO A.	Managing the Health Effects of Climate Change: Lancet and University College London Institute for Global Health Commission [32]	The Lancet
2006	McMichael A.J.	Climate Change and Human Health: Present and Future Risks [2]	Lancet
2008	Kovats R.	Heat Stress and Public Health: A Critical Review [34]	Annual Review of Public Health
2009	Anderson	Weather-Related Mortality: How Heat, Cold, and Heat Waves Affect Mortality in the United States [35]	Epidemiology
2005	Patz J.A.	Impact of Regional Climate Change on Human Health [36]	Nature
2007	Solomon S.	IPCC (2007): Climate Change: The Physical Science Basis [37]	AGU (American Geophysical Union) Fall Meeting
2007	Hoegh-Guldberg	Coral Reefs Under Rapid Climate Change and Ocean Acidification [38]	Science
2009	Basu R	High Ambient Temperature and Mortality: A Review of Epidemiologic Studies from 2001 to 2008 [39]	Environmental Health
2010	Allen C.D.	A Global Overview of Drought and Heat-Induced Tree Mortality Reveals Emerging Climate Change Risks for Forests [40]	Forest Ecology and Management
2009	Jacob D.J.	Effect of Climate Change on Air Quality [41]	Atmospheric Environment

**Table 6:** Co-occurrence frequency of the top 20 keywords.

Keywords	Frequency	Centrality	First Year
climate change	4139	0.07	2003
health	960	0.19	2003
impact	894	0.08	2003
United States	730	0.07	2003
mortality	676	0.05	2003
temperature	658	0.09	2003
air pollution	464	0.05	2003
public health	412	0.06	2005
adaptation	381	0.04	2003
human health	373	0.19	2003
vulnerability	358	0.06	2005
model	353	0.08	2003
risk	344	0.46	2003
disease	311	0.12	2004
management	311	0.02	2007
city	251	0.02	2005
variability	231	0.02	2004
exposure	206	0.02	2006
China	205	0.05	2005
heat wave	204	0.32	2003

## References

[1] Frumkin H., Hess J., Luber G., Malilay J., McGeehin M. (2008). Climate Change: The Public Health Response. Am. J. Public Health.

[2] McMichael A.J., E Woodruff R., Hales S. (2006). Climate change and human health: Present and future risks. Lancet.

[3] Ebi K.L., Ogden N.H., Semenza J.C., Woodward A. (2017). Detecting and Attributing Health Burdens to Climate Change. Environ. Health Perspect..

[4] Watts N., Adger W.N., Agnolucci P., Blackstock J., Byass P., Cai W., Chaytor S., Colbourn T., Collins M., Cooper A. (2015). Health and climate change: Policy responses to protect public health. Lancet.

[5] Thakur J.S. (2008). Protecting Health from Climate Change. Indian J. Community Med..

[6] Hsiang S., E Koppi R., Jina A.S., Rising J., Delgado M., Mohan S., Rasmusseni D., Muir-Wood R., Wilson P., Oppenheimeri M. (2017). Estimating economic damage from climate change in the United States. Science.

[7] Change I.C. (2007). IPCC, 2001: Climate Change 2001: Synthesis Report. A Contribution of Working Groups I, II, and III to the Third Assessment Report of the Intergovernmental Panel on Climate Change. http://www.ipcc.ch/publications_and_data/ar4/syr/en/contents.html.

[8] US Epa C.C.D. Human Health Impacts & Adaptation. http://www.epa.gov/climatechange/impacts-adaptation/health.html.

[9] Troeger C., Forouzanfar M., Rao P.C., Khalil I., Brown A., Reiner R.C., Fullman N., Thompson R.L., Abajobir A., Ahmed M.B. (2017). Estimates of global, regional, and national morbidity, mortality, and aetiologies of diarrhoeal diseases: A systematic analysis for the Global Burden of Disease Study 2015. Lancet Infect. Dis..

[10] Watts N., Adger W.N., Ayeb-Karlsson S., Bai Y., Byass P., Campbell-Lendrum D., Colbourn T., Cox P.M., Davies M., Depledge M. (2017). The Lancet Countdown: Tracking progress on health and climate change. Lancet.

[11] Barbara J. (2012). The impact of climate change on human health. Impact of Climate Change on Water and Health.

[12] Snow M., Snow R. (2015). The impact of climate change on human health. J. Climatol. Weather Forecast..

[13] Lichtveld M., Ouboter P., Wahid F.A., Wickliffe J., Alcala C., Zijlmans W. (2017). The impact of climate change on human health and the environment: Implications for environmental health research, policy and practice in the caribbean. West Indian Med. J..

[14] Myers S.S., Bernstein A. (2011). The coming health crisis: Indirect health effects of global climate change. F1000 Boil. Rep..

[15] Sheng R.R., Gao C.S., Chang-Chang L.I. Effects of Global Climate Change on Health of Occupational Populations: A Review. Chin. J. Public Health.

[16] Yusuf A., Francisco H. Climate Change Vulnerability Mapping for Southeast Asia. Eepsea Special & Technical Paper. https://www.preventionweb.net/go/7865.

[17] Orru H., Andersson C., Ebi K.L., Langner J., Åström C., Forsberg B. (2012). Impact of climate change on ozone-related mortality and morbidity in Europe. Eur. Respir. J..

[18] Son J.-Y., Lee J.-T., Anderson G.B., Bell M. (2012). The Impact of Heat Waves on Mortality in Seven Major Cities in Korea. Environ. Health Perspect..

[19] Jiang X.D., Wen-Qi F.U. (2008). Statistical analysis on research papers on intellectual property of digital library in China since 1996. Inf. Sci..

[20] Yao Q. (2012). A bibliometric analysis of research hotspots and research fronts on international health literacy. Chin. J. Health Educ..

[21] Small H. (1973). Co-citation in the scientific literature: A new measure of the relationship between two documents. J. Am. Soc. Inf. Sci..

[22] Wang Y. (2014). A bibliometrical analysis of status on climate change research in china based on sci database. Sci. Technol. Manag. Res..

[23] Wang B., Pan S.Y., Ke R.Y., Wang K., Wei Y.M. (2015). Erratum to: An overview of climate change vulnerability: A bibliometric analysis based on web of science database. Nat. Hazards.

[24] Pincetl S. (2015). Vulnerability studies: A bibliometric review. Prof. Geogr..

[25] Chen C. (2006). CiteSpace II: Detecting and Visualizing Emerging Trends and Transient Patterns in Scientific Literature.

[26] Liang H.-N. (2010). Overview of the Health Informatics Research Field: A Bibliometric Approach. Integr. Intern. Control Inf. Syst..

[27] Mizukami Y., Honda K., Mizutani Y., Suzuki S., Nakano J. (2017). An International Research Comparative Study of the Degree of Cooperation between disciplines within mathematics and mathematical sciences: Proposal and application of new indices for identifying the specialized field of researchers. Behaviormetrika.

[28] Chandrappa R., Kulshrestha U.C., Gupta S. (2007). Coping with climate change. Econ. Political Wkly..

[29] Peng Z., Zhang F., Zhou C., Ling Y., Bai S., Liu W., Qiu G., He L., Wang L., Wei D. (2004). Genome-Wide Search for Loss of Heterozygosity in Chinese Patients With Sporadic Colorectal Cancer. Int. J. Pancreatol..

[30] Smith D.R. (2010). Citation Analysis and Impact Factor Trends of 5 Core Journals in Occupational Medicine, 1975–1984. Arch. Environ. Occup. Health.

[31] Mustafee N., Bessis N., Taylor S.J., Sotiriadis S. (2013). Exploring the E-science Knowledge Base through Co-citation Analysis. Procedia Comput. Sci..

[32] Costello A., Abbas M., Allen A., Ball S., Bellamy R., Friel S., Groce N., Johnson A., Kett M., Lee M. (2009). Managing the health effects of climate change. Lancet.

[33] Montgomery D., Baron R., Bernstein P., Bloomberg S., Ditzel K., Lane L., Smith A., Tuladhar S., Yuan M. (2009). Impact on the Economy of the American Clean Energy and Security Act of 2009 (H.R. 2454).

[34] Kovats R., Hajat S. (2008). Heat Stress and Public Health: A Critical Review. Annu. Rev. Public Health.

[35] Anderson B., Bell M. (2009). Weather-related mortality: how heat, cold, and heat waves affect mortality in the United States. Epidemiology.

[36] Patz J.A., Campbell-Lendrum D., Holloway T., Foley J.A. (2005). Impact of regional climate change on human health. Nature.

[37] Solomon S. (2007). IPCC (2007): Climate Change The Physical Science Basis.

[38] Hoegh-Guldberg O., Mumby P.J., Hooten A.J., Steneck R.S., Greenfield P. (2007). Coral Reefs Under Rapid Climate Change and Ocean Acidification. Science.

[39] Basu R. (2009). High ambient temperature and mortality: A review of epidemiologic studies from 2001 to 2008. Environ. Health.

[40] Allen C.D., Macalady A.K., Chenchouni H. (2010). A global overview of drought and heat-induced tree mortality reveals emerging climate change risks. For. Ecol. Manag..

[41] Jacob D.J., Winner D.A. (2009). Effect of climate change on air quality. Atmos. Environ..

[42] Li H., An H., Wang Y., Huang J., Gao X. (2016). Evolutionary features of academic articles co-keyword network and keywords co-occurrence network: Based on two-mode affiliation network. Phys. A Stat. Mech. Appl..

[43] Huang C., Barnett A.G., Wang X., Vaneckova P., Fitzgerald G., Tong S. (2011). Projecting Future Heat-Related Mortality under Climate Change Scenarios: A Systematic Review. Environ. Health Perspect..

[44] Füssel H.-M., Klein R.J. (2006). Climate Change Vulnerability Assessments: An Evolution of Conceptual Thinking. Clim. Chang..

[45] Simpson M.C., Gössling S., Scott D., Hall C.M., Gladin E. (2008). Climate Change Adaptation and Mitigation in the Tourism Sector: Frameworks, Tools and Practices.

[46] Watts N., Amann M., Ayeb-Karlsson S., Belesova K., Bouley T., Boykoff M., Byass P., Cai W., Campbell-Lendrum D., Chambers J. (2018). The Lancet Countdown on health and climate change: From 25 years of inaction to a global transformation for public health. Lancet.

[47] Blake R., Corso L., Bender K. (2011). Public health department accreditation and environmental public health: A logical collaboration. J. Environ. Health.

[48] Leydesdorff L. (1997). Why words and co-words cannot map the development of the sciences. J. Assoc. Inf. Sci. Technol..

[49] Kelly P.M., Adger W.N. (2000). Theory and Practice in Assessing Vulnerability to Climate Change and Facilitating Adaptation. Clim. Chang..

[50] Berkhout F., Hertin J., Gann D. (2006). Learning to Adapt: Organisational Adaptation to Climate Change Impacts. Clim. Chang..

[51] Carrão H., Naumann G., Barbosa P. (2016). Mapping global patterns of drought risk: An empirical framework based on sub-national estimates of hazard, exposure and vulnerability. Glob. Environ. Chang..

[52] José R.S., Pérez J.L., Pérez L., Barras M.G. Effects of climate change on the health of citizens modelling urban weather and air pollution. Proceedings of the 10th International Conference on Sustainable Energy and Environmental Protection.

[53] Gosling S.N., Hondula D.M., Bunker A., Ibarreta L., Liu J., Zhang X., Sauerborn R. (2017). Adaptation to Climate Change: A Comparative Analysis of Modeling Methods for Heat-Related Mortality. Environ. Health Perspect..

[54] Sands P. (1992). The United Nations Framework Convention on Climate Change. Rev. Eur. Community Int. Environ. Law.

[55] Gu D., Yang X., Deng S., Liang  C., Wang X., Wu J., Guo J. (2020). Tracking Knowledge Evolution in Cloud Health Care Research: Knowledge Map and Common Word Analysis. J. Med. Internet Res..

